# Incidence of HTLV-1-associated myelopathy in the UK from 1991 to 2024: a longitudinal observational cohort study

**DOI:** 10.3389/fmed.2024.1519750

**Published:** 2025-01-07

**Authors:** Carolina Rosadas, Jerico Baylon, Claire Greiller, Adine Adonis, Divya Dhasmana, Nicholas W. S. Davies, Graham P. Taylor

**Affiliations:** ^1^Section of Virology, Department of Infectious Disease, Faculty of Medicine, Imperial College London, London, United Kingdom; ^2^National Centre for Human Retrovirology, St Mary’s Hospital, Imperial College Healthcare NHS Trust, London, United Kingdom

**Keywords:** HTLV-1, incidence, HAM, epidemiology, HTLV-1 associated myelopathy, tropical spastic paraparesis, proviral load, neurology

## Abstract

**Introduction:**

Human T-cell lymphotropic virus type 1 (HTLV-1) may cause spinal cord inflammation, leading to HTLV-1-associated myelopathy (HAM). HAM is a chronic and progressive neurological disorder that is associated with increased mortality and impaired quality of life. There are limited data on the incidence of HAM, with higher rates seen in Latin America and the Caribbean compared to Japan. We investigated the incidence of HAM in a cohort of initially asymptomatic people with HTLV-1 in the United Kingdom (UK).

**Methods:**

This is a longitudinal retrospective observational study of people with confirmed HTLV-1 infection who first attended the National Centre for Human Retrovirology in London, UK, between 1st March 1991 and 31st March 2024. Clinical records were analysed to calculate the incidence rate and cumulative incidence of HAM. A secondary analysis was conducted to assess HAM incidence in people living with HTLV-1 and HIV coinfection. At the first visit, the HTLV-1 proviral load was compared between incident cases and those who remained asymptomatic during follow-up.

**Results:**

In a cohort with up to 33 years of follow-up of individuals living with HTLV-1 in the UK, the cumulative incidence of HTLV-1-associated myelopathy (HAM) was found to be 1.35% (4/297), with an incidence rate of 1.98 per 1,000 person-years. All people who developed HAM had a high proviral load at the first clinic visit. No cases of incident HAM were observed among individuals who had HIV-HTLV-1 coinfection during the study period.

**Discussion:**

The incidence of HAM in the UK cohort is comparable to the rates reported in Latin America and the Caribbean and is higher than reported in other high-income countries. High proviral load of HTLV-1 predates the development of HAM.

## 1 Introduction

The human T-cell lymphotropic virus type 1 (HTLV-1) is a blood-borne sexually and vertically transmitted virus discovered in 1980, with at least 5–10 million individuals infected worldwide (1). In 1985, HTLV-1 was strongly associated with tropical spastic paraparesis (TSP), a neurological disease of unknown aetiology that was reported in Latin America and the Caribbean. At the same time, HTLV-1-infected cells were detected in the cerebrospinal fluid of patients with spinal spastic paraparesis in Japan, leading to the identification a novel entity, HTLV-1-associated myelopathy (HAM). The research community soon recognised TSP and HAM as a single clinical entity, with HTLV-1 identified as the aetiological agent (2). HAM causes long-term disability, with symptoms including urinary urgency/incontinence, pain—particularly in the lower back and limbs—and sexual dysfunction, in addition to spastic paraparesis. Disease progression in the majority of patients is slow, but half of the patients are wheelchair-dependent (3) 20 years after disease onset. Rapid progression occurs in 15% of cases, with at least a unilateral walking aid required within 2 years of symptom onset (4). The quality of life (QoL) of patients with HAM is poor, with those from the UK scoring the lowest QoL, as assessed using the EQ-5D - EuroQol - 5-Dimensions-index, compared to more than 130 other medical conditions (5). In a multicentre study conducted in Brazil and the UK, one out of every five individuals with HAM reported that their quality of life was worse than death (5). Recent data from several countries, including Japan, Brazil, and the UK, indicate that patients with HAM have a higher risk of mortality compared to those living with HTLV-1 asymptomatic infection (6, 7). Despite HTLV-1 being known for more than four decades now, data on HAM epidemiology remain scarce. However, higher disease incidence is reported in Latin America and the Caribbean compared to Japan (8–12). In the HTLV-1 technical report, published in 2020, the World Health Organization recommended conducting cohort studies to provide insights into geographical differences in the manifestation and progression rates of HTLV-1-associated diseases and to calculate the burden of disease, helping countries understand the impact of HTLV-1 infection and enabling the implementation of effective policies (1). This study aimed to determine the incidence of HAM in a cohort of individuals living with HTLV-1 in the United Kingdom (UK) over more than 30 years of follow-up (1991–2024).

## 2 Methods

### 2.1 Standard protocol and ethics

All data were originally collected as part of routine clinical management and were then anonymised for analysis under the regulations of the National Research Ethics Service. This study adhered to the STROBE - The strengthening the Reporting of Observational Studies in Epidemiology-guidelines (13).

### 2.2 Cohort description, outcomes, and statistical analysis

In the early 1990s, our group established a national reference service for individuals living with HTLV-1 and 2. This service is known as the National Centre for Human Retrovirology (NCHR) and is located at St Mary’s Hospital, Imperial College Healthcare NHS Trust in London, UK. All patients are offered life-long follow-up with regular reviews by a multidisciplinary team, and prospective recordings of detailed clinical and laboratory data are available. For this longitudinal retrospective analysis, only people with confirmed HTLV-1 infection who first attended after 1st March 1991, were asymptomatic at their first visit, and had at least one year of follow-up by 31st March 2024 were included. HTLV-1 infection was confirmed serologically using the Western Blot method (HTLV Western Blot 2.2 and 2.4, Genelabs) at the National Reference Laboratory (UK Health Security Agency, Colindale, London) and/or by the molecular detection of the HTLV-1 proviral genome in peripheral blood mononuclear cells (Molecular Diagnostic Unit, Imperial College London) (14). The exclusion criteria included individuals with proven *Strongyloides stercoralis* coinfection, children (<16 years of age), and people living with HTLV-2. Individuals coinfected with HIV-1 were analysed as an independent group (secondary analysis). HAM was diagnosed according to an international consensus known as the Castro-Costa criteria (15). According to these criteria, definite HAM cases are characterised as follows: (1) A non-remitting progressive spastic paraparesis with a sufficiently impaired gait to be perceived by the patient. Sensory symptoms or signs may or may not be present. When these symptoms are present, they remain subtle and lack a clear sensory level. Additionally, urinary and anal sphincter symptoms may or may not be present; (2) The presence of HTLV-I antibodies in serum and CSF confirmed through methods such as Western blotting and/or a positive PCR test for HTLV-I in blood and/or CSF; and (3) Exclusion of other disorders that can resemble TSP/HAM. In April 2024, an anonymised clinical database was accessed, and the relevant information was exported [gender, ethnicity, date of birth, date of first visit, date of last visit, and HTLV-1 proviral load (PVL) in peripheral blood mononuclear cells (PBMCs) at the baseline visit]. The HTLV-1 PVL is measured through real-time PCR and it is determined by calculating the number of copies of HTLV-1 Tax gene per 200 copies of the beta-globin gene and is expressed as HTLV-1 DNA copies per 100 PBMCs (or as a percentage) (14). The primary outcome was the incidence rate, calculated as the number of incident cases divided by the number of people living with HTLV-1 asymptomatic infection multiplied by follow-up time (in years). The cumulative incidence was determined as the ratio of incident HAM (iHAM) to the number of individuals living with HTLV-1 included in the study. A 95% confidence interval is presented. A subgroup analysis by gender was performed. The Fisher exact test was used to compare proportions. A secondary analysis was performed to determine HAM incidence in people living with HTLV-1-HIV coinfection.

## 3 Results

**Figure 1:**
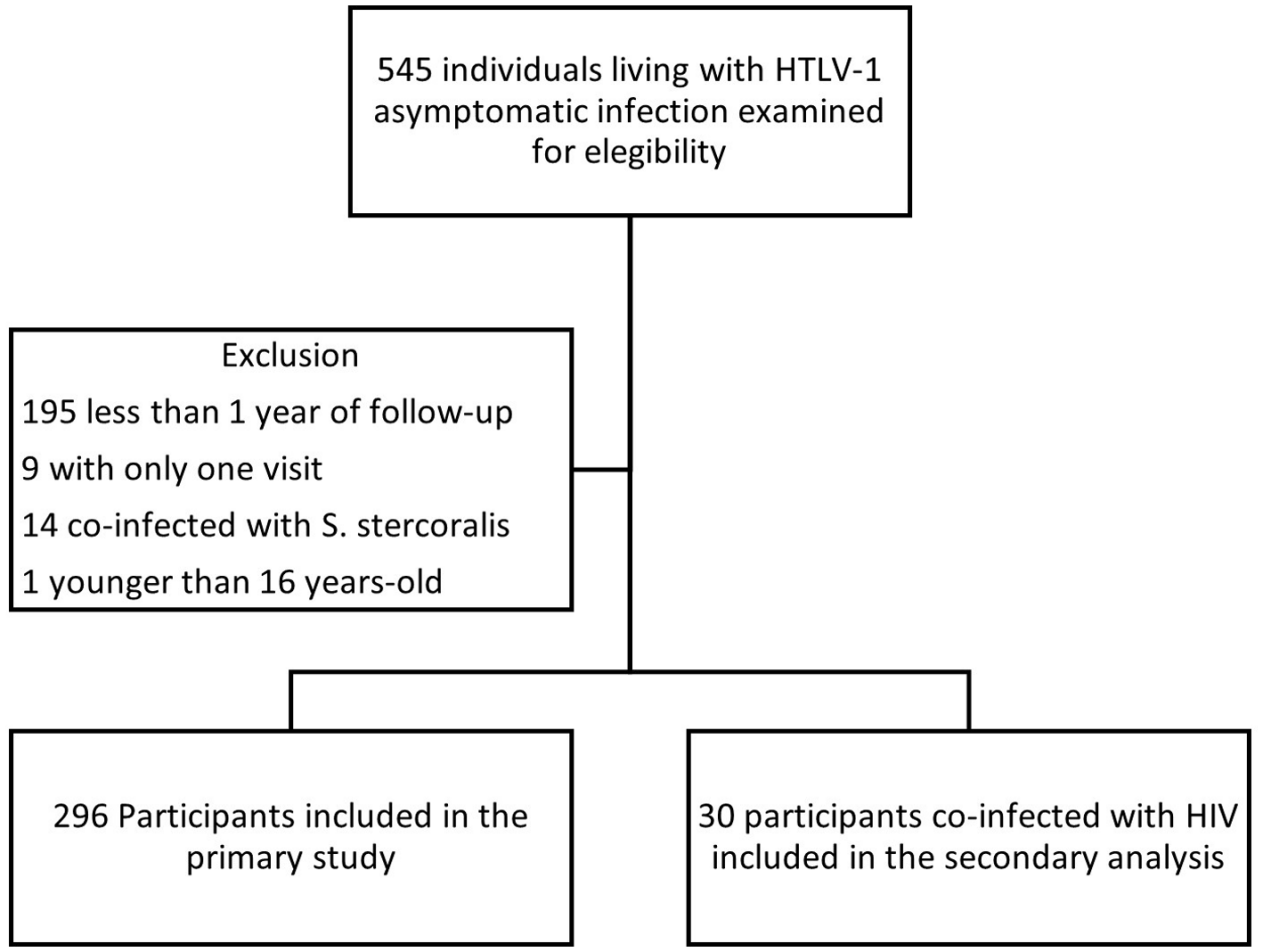
Flow diagram of study participants.

Of the 545 individuals living with HTLV-1 asymptomatic infection at the time of the first clinic visit, 219 were excluded (195 had less than 1 year of follow-up, 9 had only one visit, 14 were coinfected with *S. stercoralis*, and 1 was younger than 16) (Figure 1). A total of 296 participants were included in the primary analysis (comprising 70 [24%] male individuals and 226 [76%] female individuals). Over half of the cohort participants (51%) were Afro-Caribbeans, whereas only 16.5% were Caucasians. The mean age of the participants at baseline was 46.8 years (range 17–86 years) (Table 1). The total follow-up was 2,022 person-years, with a mean of 6.8 years (range 1 to 31.1 years). A total of 69.2% of subjects had more than 3 years of follow-up and 53% had more than 5 years. The remaining 30 patients were co-infected with HIV-1 and were analysed separately.

**Table 1:** General characteristics of the study participants.

Sex % (n)	
Male	24 (70)
Female	76 (226)
Age at baseline Mean (Range)	46.8 years (range 17–86 years)
Ethnicity/Origin % (n)	
Afro-Caribbean	51 (151)
Caucasian	16.5 (49)
Other	32.5 (96)
Asia	*n* = 46
Africa	*n* = 29
South America	*n* = 6
Mixed	*n* = 6
Unknown	*n* = 9
Follow-up duration (years) Mean (range)	6.8 (1–31.1)

During the follow–up period, four patients developed HAM, resulting in a cumulative incidence of 1.35% (95% CI: 0.4–3.55%). All incident cases were among female patients, leading to an adjusted cumulative incidence of 1.77% (95% CI: 0.53–4.62%) for female individuals and 0% (95% CI: 0–6%) for male individuals (*p* > 0·05). The age range at which HAM onset occurred ranged from 39 to 67 years, whilst the time since the start of follow-up to disease onset varied from 7 months to 6 years. Two women who developed HAM were Afro-Caribbean, one was Caucasian, and another originated from Iran (Table 2).

**Table 2:** Description of incident HAM cases.

	iHAM 1	iHAM 2	iHAM 3	iHAM 4
Sex	F	F	F	F
Age at HAM onset (years)	39	67	63	59
Time since start of follow-up to disease onset	3.5 years	6 years	4 years	7 months
HTLV-1 PVL in PBMC at baseline	1.4%^*^	34.6%	8.12%	2.3%
Ethnicity/Country of origin	Caucasian	Afro-Caribbean	Iranian	Afro-Caribbean

* Proviral load estimated based on limiting dilution. F, Female; iHAM, incident HAM; PVL, HTLV-1 Proviral load; PBMC, Peripheral blood mononuclear cells.

**Figure 2:**
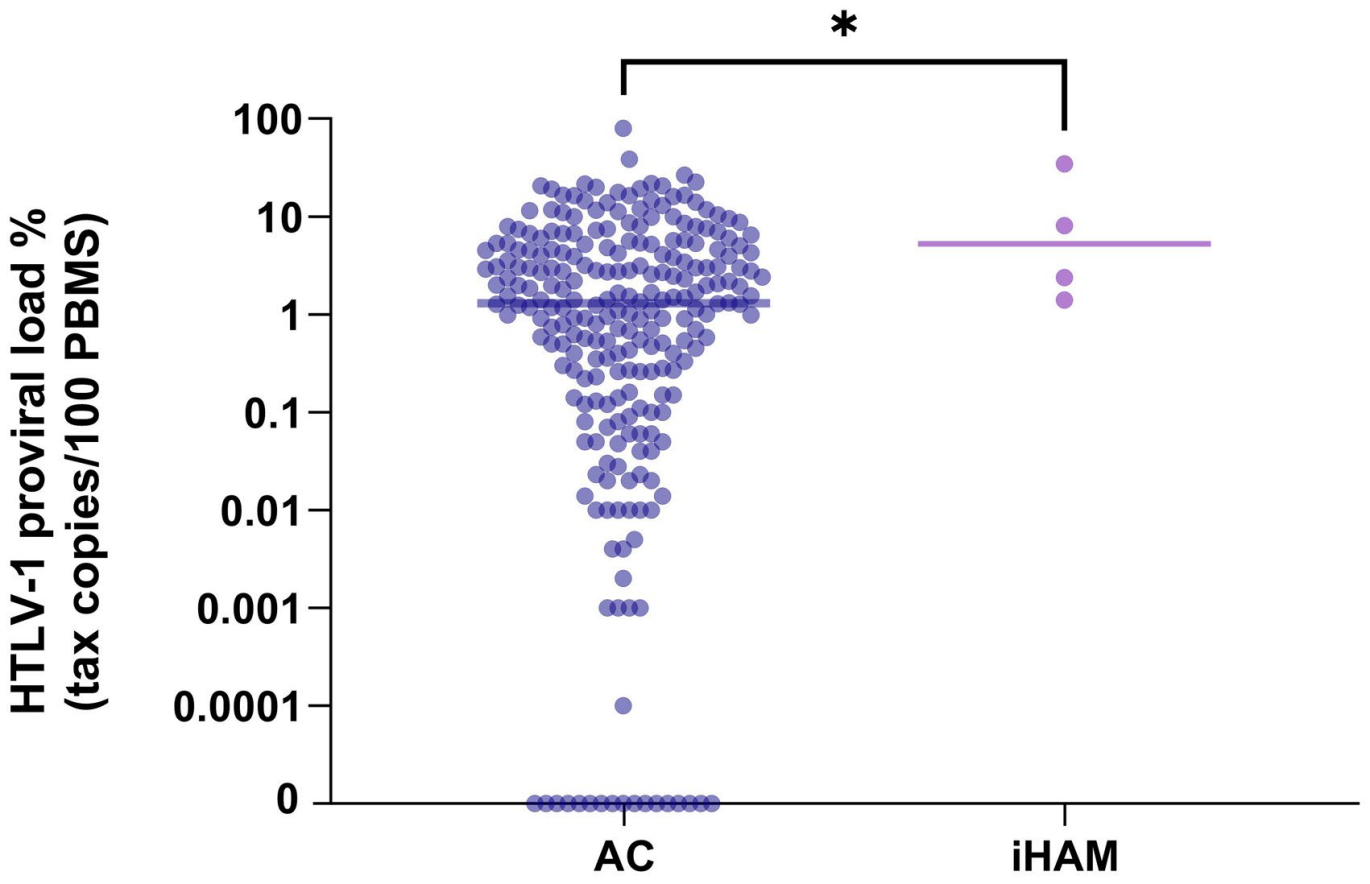
HTLV-1 proviral load in asymptomatic carriers who did not develop HAM compared with those with incident HAM.

During the analysis period (1991–2024), the incidence rate was 1.98 per 1,000 person-years (95% CI: 0.6–5.4). The HTLV-1 proviral load in PBMCs at baseline was higher in individuals who developed iHAM compared to those who remained without HAM during the follow-up (Median [Min-Max]: 5.2% (1.4–34.6%) versus 1.32% (0–79.6%); *p* = 0.04) (Figure 2).

There were 30 additional cases of HIV-1 and HTLV-1 coinfection, consisting of 14 male and 16 female individuals. Among these, 16 were Afro-Caribbean, 11 were originally from Africa, 1 had mixed origin, and 2 were Caucasian. Their mean follow-up duration was 7.5 years (ranging from 1.3 to 22.5 years) and the mean age at the first clinic visit was 48.2 years (ranging from 28 to 68 years). iHAM was not observed in this group during 224.7 person-years of follow-up (95%CI: 0/1,000 person-years to 0.075/1000 persons-years). The male-to-female ratio was higher in the coinfected group compared to the HTLV-1 mono-infected group (*p* = 0.0142).

## 4 Discussion

This is the first report of HAM incidence during the long-term follow-up of a cohort of individuals living with HTLV-1 in a non-endemic area. The incidence of 1.35% is similar to that of Brazil and the Caribbean. In Salvador, Brazil, a cumulative incidence of 1.47% (5/251) was observed during a median of 4 years of follow-up (1–7 years) (8). In Minas Gerais, Brazil, the cumulative incidence reached 3.9% (7/181) in a cohort that was followed for a mean of 7 years (1997–2011), with an incidence density of 5.3 cases per 1,000 HTLV-1-seropositive cases per year (95% CI: 2.6–10.9) (11). Similar results were reported in São Paulo, Brazil, with an incidence of 5.9% (12/204) during 5 years of follow-up (16). In the US, follow-up of 136 blood donors living with HTLV-1 over 2 years revealed no cases of HAM (17). Maloney et al. reported an overall lifetime risk of developing HAM of 1.9% in Jamaica (10). In Martinique, the crude incidence rates of HAM per 100,000 during each 5-year period (95%CI) were 10.01 (6.78–13.28, 1986–1990), 13.02 (9.34–16.70, 1991–1995), 11.54 (8.13–14.95, 1996–2000), 4.27 (2.24–6.28, 2001–2005), and 2.03 (0.62–3.43, 2006–2010) (9). In Japan, a lifetime risk of developing HAM was estimated to be 0.25% in 1982–1988 (12). In the three later studies, the authors extrapolated the number of individuals living with HTLV-1 using data from seroprevalence studies and census information (10, 12). The similarities between our findings and data from South America and the Caribbean may be explained by the fact that the majority of the people with HTLV-1 in the UK are predominantly of Caribbean and West African heritage (high prevalence areas, with higher HAM incidence).

Interestingly, the age-standardised 5-year incidence rates from Martinique indicates a significant decrease over time (69% in 2001–2005 and 87% in 2006–2010, when compared to the rates recorded in 1986–1990) (9). The authors hypothesised that this decline could be attributed to interventions implemented in the early 1990s, including screening of pregnant women and blood and organ donors, and awareness campaigns about sexually transmitted infections, including HTLV, which promoted safe sex practices (9). This successful experience encourages the implementation of similar interventions in other settings to reduce the burden of HTLV-1-associated diseases. This is increasingly significant as the influx of migrants from high-endemic areas to low-endemic areas continues to rise. Screening for HTLV-1 in migrants is recommended in the UK (18); however, it is rarely performed (19). Similar scenarios are observed in other European countries, such as Spain, where 2–3 new cases of HAM are reported every year, despite limited availability of HTLV-1 testing and a lack of awareness about the virus (20).

HAM is reported to be more common in women. In Jamaica, the age-standardised HAM incidence rate was higher among women (24.7/100,000 PY) compared to men (17.3/100,000 PY) (10). In this study, all iHAM cases were among female patients, but the difference was not statistically significant because women predominated amongst those living with HTLV-1 in our cohort. This can be partially attributed to the higher efficiency of sexual transmission from male to female than from female to male; however, other factors selectively lead to the greater likelihood of testing women in the UK. For example, UK blood donors are predominantly female individuals, whilst male contacts are less likely to attend for testing.

In those who later developed HAM, a higher HTLV proviral load at the first clinic visit was observed. This is in agreement with the published literature, confirming that a high proviral load is a risk factor for HAM (21). It is important to note that, as observed in our cohort, people with asymptomatic infection may also possess a high proviral load. Therefore, a high proviral load appears to be necessary but not sufficient alone to cause HAM. Quantification of the proviral load appears to be useful to reassure patients who have a low risk of developing disease and to identify those who would benefit from more frequent clinical reviews and additional clinical, laboratory, and/or image investigations.

One limitation of the study is that the time of infection is not known. Therefore, the duration of follow-up was used instead of the duration of infection. Another limitation is the number of patients with a follow-up of less than 1 year.

In our study, we focused our analysis on instances of incident HAM that fulfilled the definitive diagnostic criteria, as established by experts in 2006 (Castro Costa diagnostic criteria) (15). However, it is now becoming increasingly acknowledged that the impact of HTLV-1 inflammation is not limited to HAM. A significant proportion of patients with neurological symptoms (not fulfilling HAM diagnostic criteria) has been reported in different cohorts, particularly from Brazil, reaching up to 30% of those living with the virus (8, 22). Furthermore, we have noted that a proportion of asymptomatic individuals in the UK have activated lymphocytes with evidence of subclinical neuronal damage, demonstrated by high plasma levels of neurofilament light chain (23). In addition to neuroinflammation, multisystemic inflammation may also be observed in people with HTLV, and it is not restricted to those with HAM. Uveitis, dermatitis, arthritis, polymyositis, and lung inflammation are also known outcomes of HTLV-1 infection (24). Therefore, the impact of HTLV-1 is even greater, and chronic inflammation may contribute to the reported 1.57-fold increase in mortality associated with HTLV-1 infection (24).

Although data indicate a higher risk of HAM in people with HIV-HTLV-1 coinfection compared to those with HTLV-1 single infection, there were no incident cases of HAM in the subgroup analysis of the present study. The limited number of participants with HIV-HTLV-1 coinfection and the higher proportion of males in this group may have influenced the results and preclude definitive conclusions.

The cumulative incidence of HAM in the UK is similar to that observed in other countries, particularly Latin America and the Caribbean, and is higher than that reported in Japan. There are no data on HAM incidence in Africa, where HTLV infection is poorly described, despite high prevalence reported in many countries of West, Central, and Southern Africa (25). The mechanisms that determine the incidence of HAM are unclear. High proviral load is a prerequisite, but it is insufficient alone. Studies to determine what triggers increased passage of HTLV-1-infected cells across the blood–brain–barrier and provokes an inflammatory response in central nervous system the are required to understand and prevent HAM in people living with HTLV-1. Case–control studies on the incidence of other inflammatory conditions associated with HTLV-1 would be beneficial to improve our understanding of the all-encompassing impact of HTLV-1 infection. Interventions aimed at limiting the spread of HTLV-1 are encouraged, as they will reduce the incidence of HTLV-1-associated diseases over time.

## Data Availability

The raw data supporting the conclusions of this article will be made available by the authors, without undue reservation.
